# Production, Optimization and Characterization of Biobased Itaconic Acid from *Saccharomyces cerevisiae*

**DOI:** 10.3390/ijms27198923

**Published:** 2026-10-08

**Authors:** Zulfiye Velioglu Tosuner, Mehdi Kosaca, Raziye Ozturk Urek, Onur Yilmaz, Catalina Natalia Yilmaz

**Affiliations:** 1 Science and Technology Application and Research Center, Dokuz Eylul University, 35390 Buca, Türkiye; zulfiye.velioglutosuner@deu.edu.tr; 2 Biochemistry Division, Department of Chemistry, Faculty of Science, Dokuz Eylul University, 35390 Buca, Türkiye; kosacamehdi@gmail.com (M.K.); raziye.urek@deu.edu.tr (R.O.U.); 3 Biotechnology Department, The Graduate School of Natural and Applied Sciences, Dokuz Eylul University, 35390 Buca, Türkiye; 4 Leather Engineering Department, Faculty of Engineering, Ege University, 35100 Bornova, Türkiye; onur.yilmaz@ege.edu.tr

**Keywords:** itaconic acid, *Saccharomyces cerevisiae*, submerged fermentation, characterization, polymerization

## Abstract

Itaconic acid (IA) is an important bio-based platform chemical with applications in the production of polymers, coatings, resins, and other value-added materials. However, its conventional microbial production relies predominantly on microorganisms that present safety and process-related limitations. This study investigated *Saccharomyces cerevisiae* BY4742 as a potentially safer microbial platform for the production of bio-based IA under submerged fermentation conditions. IA production was optimized using response surface methodology with a Box–Behnken design, considering glucose concentration, initial pH, and CaCI_2_ concentration as independent variables. The maximum IA concentration of 1133.50 ± 54.92 mg/L was obtained at 30 °C, pH 4.5, with 10% (*w*/*v*) glucose and without CaCI_2_ supplementation after 216 h of fermentation. The fermentation-derived IA was subsequently recovered and characterized using high-performance liquid chromatography, Fourier-transform infrared spectroscopy, ^1^H-nuclear magnetic resonance (^1^H-NMR), and thermogravimetric analysis. The analytical results confirmed the molecular identity and high chromatographic purity of the recovered IA. Furthermore, the homopolymerization ability of the fermentation-derived IA was evaluated and compared with that of commercial IA under the same reaction conditions. The ^1^H-NMR results demonstrated changes consistent with consumption of the IA C=C double bond following polymerization, indicating that the fermentation-derived IA retained its ability to undergo homopolymerization. Overall, these findings demonstrate the potential of *S. cerevisiae* for producing structurally confirmed bio-based IA and provide a proof of concept for its use as a renewable monomer for poly(itaconic acid) synthesis, supporting its further investigation for the development of bio-based polymeric materials.

## 1. Introduction

Itaconic acid (IA) is an important bio-based platform chemical that can serve as a building block for the production of polymers, coatings, resins, and other value-added materials. Its importance was first highlighted by the U.S. Department of Energy, which identified IA among the top 12 value-added chemicals that can be produced from biomass [1]. More recent studies have continued to recognize IA for sustainable polymer production [2,3]. Owing to its molecular structure, which contains a reactive C=C double bond and two carboxylic acid groups, IA is a versatile monomer for polymer synthesis and can be used in the development of materials for applications including synthetic latexes, superabsorbent polymers, paints, textiles, and biomedical products [4,5]. As market demand continues to grow, with the global IA market estimated at approximately USD 117–120 million in 2025 [6], the development of efficient microbial fermentation processes for IA production from renewable resources has attracted increasing attention.

IA is mainly produced by the fungus *Aspergillus terreus* [7], and this microorganism has been employed for IA production for many years. However, the use of *A. terreus* presents several process-related challenges. During fermentation, *A. terreus* develops filamentous growth, which can increase culture viscosity and complicate mixing, potentially limiting oxygen transfer and making process control more difficult [8]. In addition, *A. terreus* is considered an opportunistic pathogen microorganism and have been associated with the production of tremorgenic mycotoxins capable of inducing tremor in mammals, with effects ranging from intermittent to persistent symptoms [9]. The species has also been reported to cause disease in plants. Consequently, its use for industrial IA production requires appropriate biosafety measures and careful process control [10]. These characteristics may increase process complexity and limit the practical advantages of large-scale fermentation. To address these challenges, alternative microorganisms, particularly yeasts, have been investigated for IA production. In recent years, yeast species with established applications in food and biotechnology have received increasing attention as alternative microbial platforms for IA production. Several studies have demonstrated the potential of *Yarrowia lipolytica* for IA production [11], while metabolic engineering approaches have also been employed in other yeast systems to enhance IA biosynthesis [12]. Another promising microorganism is *Saccharomyces cerevisiae*, a well-established model and industrial yeast with a long history of use in food and biotechnology applications. Unlike filamentous fungi, *S. cerevisiae* grows predominantly as individual cells, which can facilitate mixing and oxygen transfer during cultivation [13]. In addition to microorganism selection, both the operational conditions and the composition of the cultivation medium are critical factors affecting IA production. Alongside carbon availability, initial pH, trace element concentrations, and operational conditions (temperature, agitation, etc.) comprehensively influence microbial growth, metabolic activity, and final product yield.

Among the carbon sources investigated for microbial IA production, including monosaccharides, hydrolyzed sugars, agro-industrial residues, and polymeric carbohydrate substrates, glucose has frequently been reported as an effective substrate for IA biosynthesis [14,15,16]. The initial pH of the production medium is another important parameter because it can affect both microbial metabolism and the recovery of IA from the fermentation broth. Although acidic conditions favor the protonated form of IA and may facilitate downstream recovery, excessively low pH values can negatively affect microbial activity by increasing cellular stress and product inhibition [17]. In addition, metal ions can influence IA production through their effects on enzymatic activity, cellular metabolism, and transport processes [18]. Among the metal ions investigated in IA-producing systems, manganese, iron, copper, zinc, and calcium have been shown to influence production depending on the microorganism and cultivation conditions [8]. Therefore, systematic optimization of these cultivation parameters is essential for improving IA production and establishing an efficient fermentation process.

In this study, *S. cerevisiae* BY4742 was employed for IA production under submerged fermentation (SmF) conditions. To optimize the fermentation parameters, response surface methodology (RSM) was employed. Compared to the traditional one-factor-at-a-time approach, which is time-consuming and incapable of detecting interactions between variables, RSM is a powerful mathematical and statistical tool that reduces the number of required experimental runs and effectively evaluates the interactive effects among multiple parameters [19]. Specifically, RSM using a Box–Behnken design (BBD) was applied to systematically evaluate the effects of glucose concentration, initial pH, and CaCI_2_ concentration on IA production and to determine the combination of conditions producing maximum IA production. Following fermentation, the produced IA was recovered and characterized using high-performance liquid chromatography (HPLC), Fourier-transform infrared spectroscopy (FT-IR), ^1^H-nuclear magnetic resonance (^1^H-NMR), and thermogravimetric analysis (TGA), and derivative thermogravimetric analysis (DTG) to confirm its molecular identity and evaluate its physicochemical properties. Finally, the newly obtained fermentation-derived IA (f-IA) was subjected to homopolymerization under the selected reaction conditions and compared with commercial IA (c-IA) to evaluate its polymerization ability and assess its potential as a renewable monomer for further polymer synthesis. The resulting polymers were designated f-PIA and c-PIA, respectively.

The novelty of this study lies in its comprehensive evaluation of *S. cerevisiae* for IA production under SmF conditions, including process optimization, product recovery and characterization, and assessment of the polymerization capability of the f-IA. Furthermore, the use of the non-pathogenic bakery yeast *S. cerevisiae* and the demonstrated recovery and polymerization potential of the produced f-IA provide a basis for further investigation and optimization of bio-based IA monomer and its potential application.

## 2. Results

### 2.1. Optimization of IA Production

**Figure 1:**
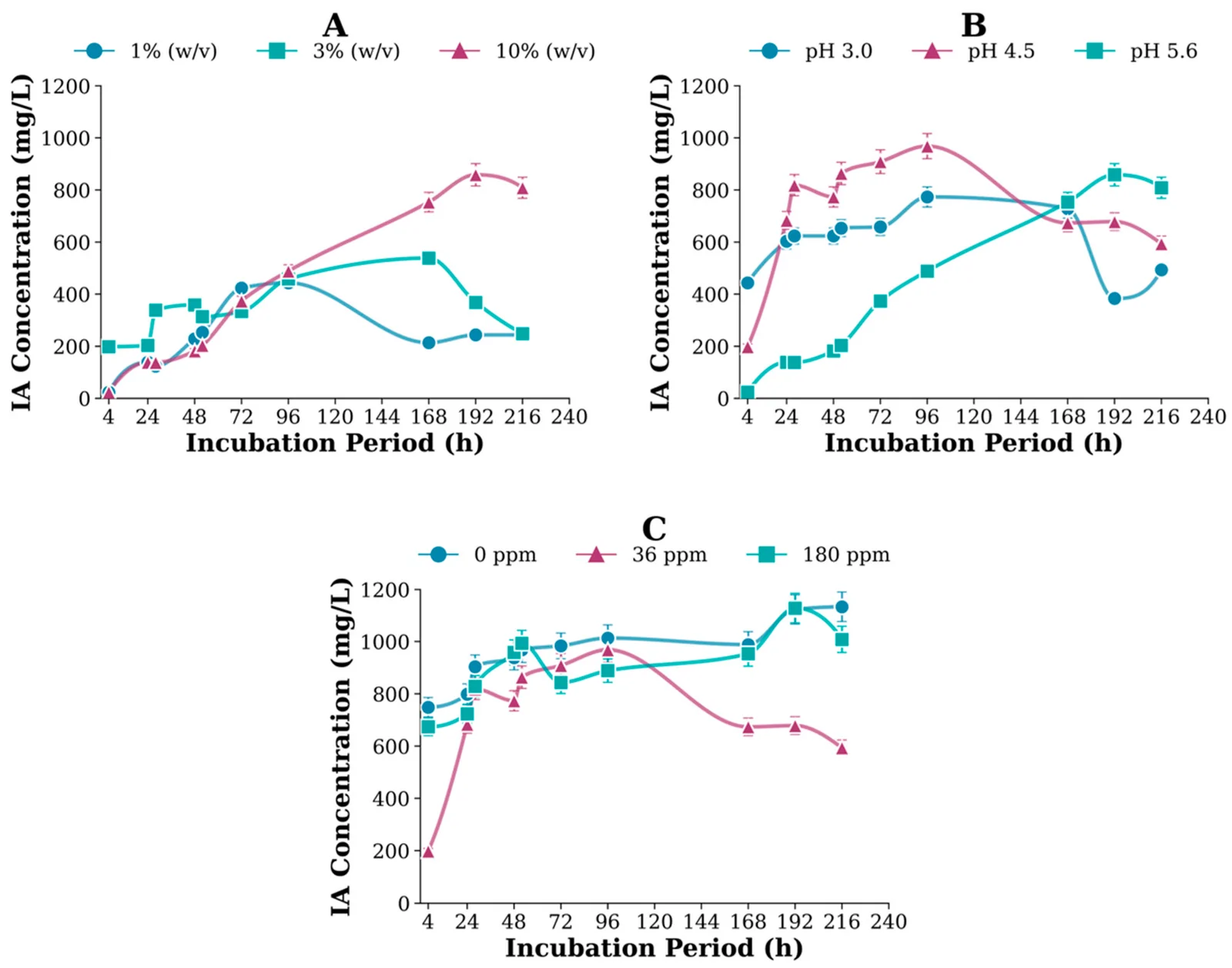
f-IA concentration through the incubation period in production media: (**A**) with different D-glucose concentrations (*w*/*v*), (**B**) with different initial pH values with 10% (*w*/*v*) D-glucose concentration, and (**C**) with different CaCl_2_ concentrations with 10% (*w*/*v*) D-glucose concentration and pH 4.5.

In the present study, *S. cerevisiae* was cultivated in different media containing varying concentrations of D-glucose (1, 3 and 10%, *w*/*v*), initial pH levels (3, 4.5 or 5.6) and CaCl_2_ concentrations (0, 0.01, 0.05 g/L). The produced f-IA content was monitored spectrophotometrically throughout the incubation period (Figure 1). During the initial 96 h of incubation, no significant difference was observed in f-IA production among the media containing different glucose concentrations. However, at the later stages of incubation, the medium supplemented with a higher concentration of carbon source exhibited greater f-IA production compared to the other media. The highest f-IA concentration (858.50 ± 41.18 mg/L) was detected in 10% (*w*/*v*) D-glucose medium on the 192 h of incubation period (Figure 1A). In the subsequent stages of the study, 10% glucose was used as the carbon concentration, and the effect of the initial pH of the incubation medium on f-IA production was investigated.

Evaluation of the fermentation results obtained under acidic pH conditions revealed that the highest f-IA production (968.50 ± 47.25 mg/L) was achieved in the medium with an initial pH value of 4.5 on the 96 h of incubation (Figure 1B). In the final stage of the optimization study, the effect of CaCl_2_ concentration in the production medium on f-IA production was investigated (Figure 1C). Under the optimized conditions (10% (*w*/*v*) D-glucose, pH 4.5, calcium deficiency), the maximum f-IA concentration reached 1133.50 ± 54.92 mg/L after 192 h of incubation. The value obtained at 216 h of fermentation (1138.50 ± 55.36 mg/L) was not significantly different from the value obtained at 192 h. Compared with the maximum f-IA concentration obtained during the initial glucose optimization step (858.50 ± 41.18 mg/L), the optimized process increased f-IA production by approximately 32.03%.

**Figure 2:**
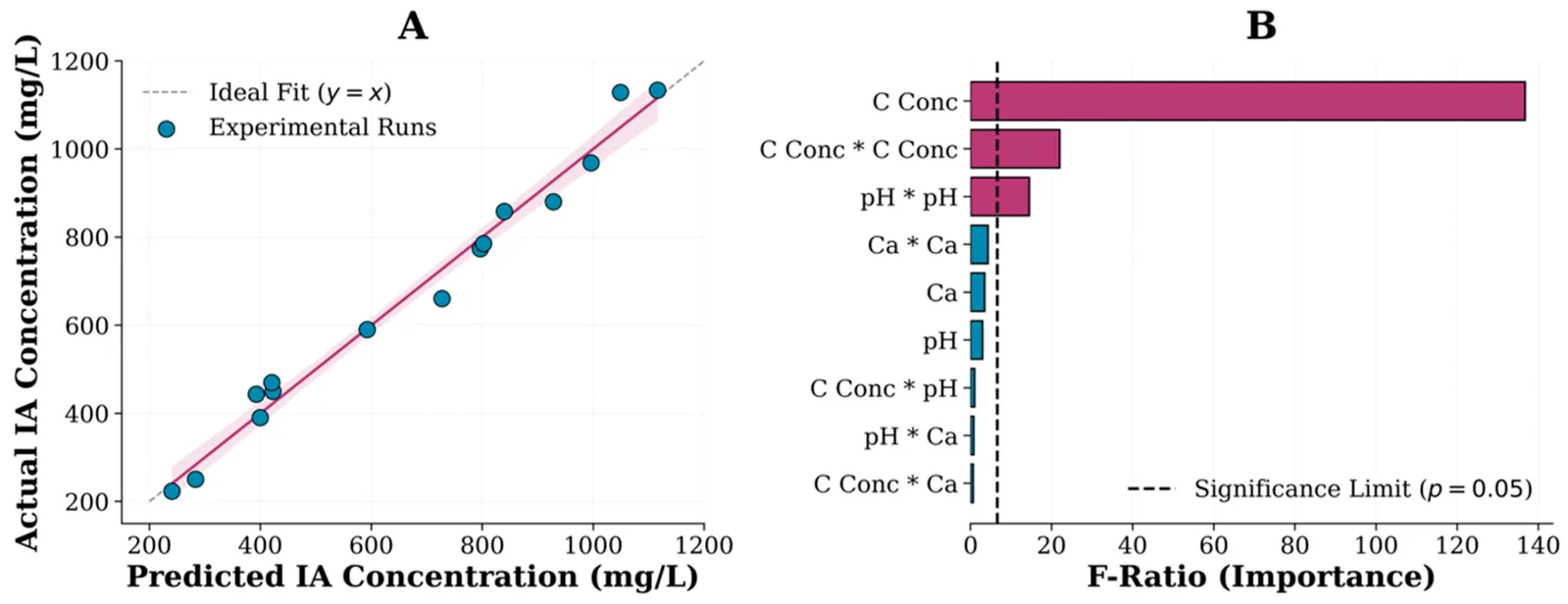
(**A**) Correlation between the actual and predicted f-IA levels (R^2^ = 0.9816). (**B**) Pareto chart showing the standardized effects of independent variables on f-IA production. The red dashed line represents the statistical significance limit (*p* = 0.05).

To maximize f-IA production, the combined effects of three independent variables—carbon concentration (1–10%), pH (3.0–5.6), and CaCI_2_ concentration (0–0.05 g/L)—were investigated using an RSM. A total of 15 experimental runs were conducted, and the actual IA production yields were compared with the values predicted by the quadratic mathematical model (Figure 2A).

The statistical analysis (ANOVA) confirmed that the quadratic model was highly significant and reliable (*F*-value = 29.59, *p* = 0.0008). The determination coefficient (R^2^) was found to be 0.9816, with an adjusted R^2^ of 0.9484 and a predicted R^2^ of 0.8122, indicating that the model robustly explains the variation in f-IA production and possesses strong predictive capability. Although a formal lack-of-fit test could not be calculated due to absence of exact replicate points in the design matrix to estimate pure error, the strong correlation metrics and high predictive capability demonstrate that the quadratic model fits the experimental data well and is adequate for navigating the design space. The detailed statistical analysis results of the BBD, including coefficient estimates, *t*-values, and *p*-values for each linear, quadratic, and interaction term, are summarized in Table 1. As shown in Figure 2A, the experimental data points fit closely to the ideal prediction line, indicating an excellent agreement between the observed and predicted results. As shown in Figure 2A, the experimental data points fit closely to the ideal prediction line, indicating an excellent agreement between the observed and predicted results.

To understand which variables had the most significant impact on f-IA production, a Pareto chart was generated (Figure 2B). According to the Pareto chart, the linear effect of carbon concentration was the most dominant factor controlling f-IA production, followed by the quadratic effects of carbon concentration and initial pH. Since these bars cross the red significance line, they are statistically significant for the fermentation process. In contrast, the linear effect of CaCl_2_ concentration and its interactions with other variables did not show a statistically significant effect on the final f-IA production.

**Figure 3:**
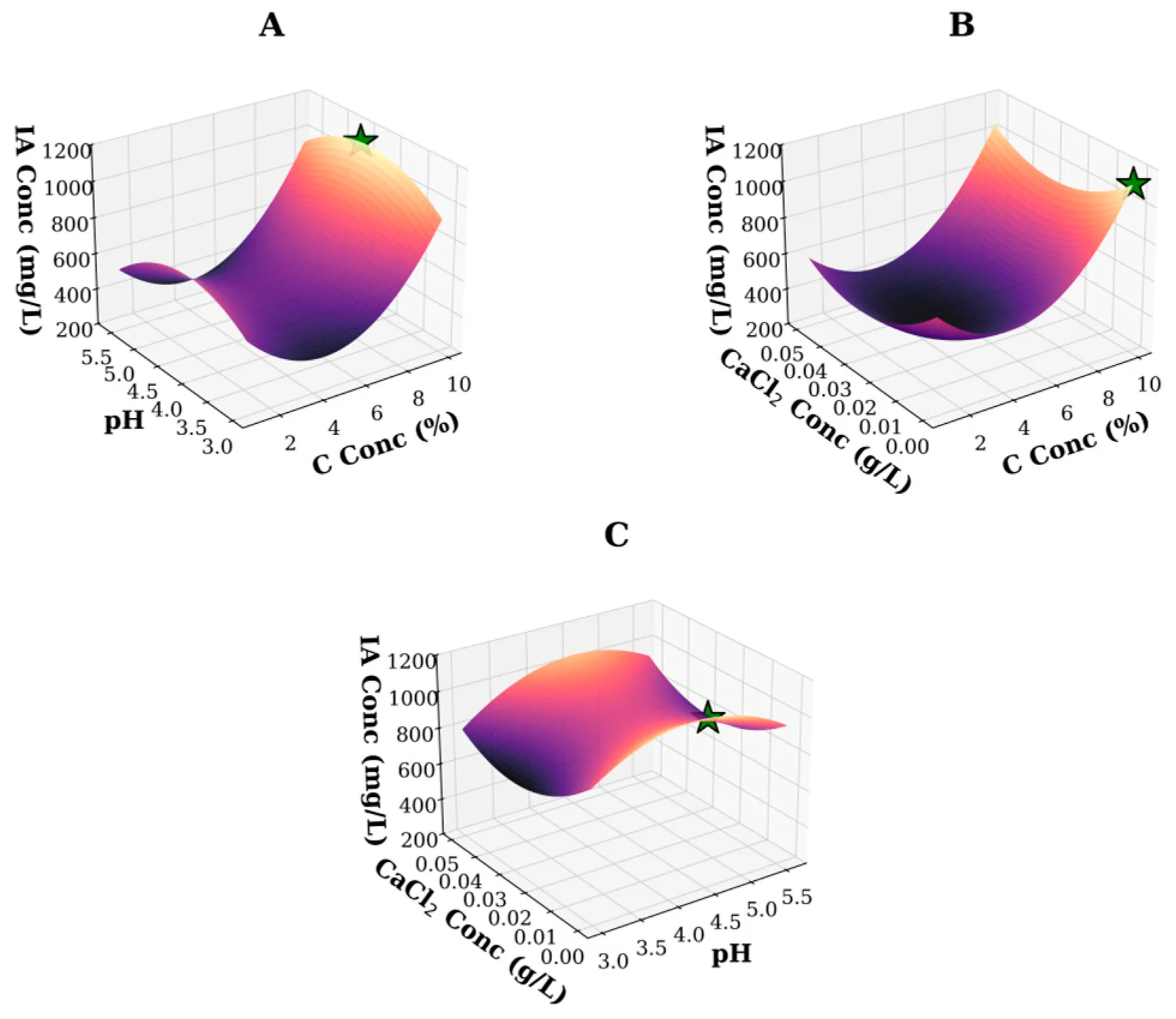
3D response surface and 2D contour plots showing the interactive effects of; (**A**) Carbon concentration and pH, (**B**) Carbon concentration and CaCI_2_ concentration, (**C**) pH and CaCl_2_ concentration on f-IA production. The green star indicates the theoretical optimum point.

To visually determine the optimum conditions, 3D response surface plots and 2D contour maps were generated (Figure 3). These plots show how f-IA level changes when two variables are modified simultaneously while keeping the third variable constant at its optimum level.

The 3D surface plots clearly demonstrate that increasing the carbon concentration up to 10% drastically improves f-IA production. Similarly, a moderate pH level (around 4.5) provided the highest product accumulation. Based on the mathematical model, the maximum f-IA production (1116.14 mg/L) was predicted under the optimum conditions of 10% (*w*/*v*) glucose, an initial pH of 4.5, and 0 g/L CaCl_2_ addition. The confirmatory experiments were evaluated under these optimized conditions, resulting in an actual maximum IA concentration of 1133.50 ± 54.92 mg/L (as also observed in Figure 1C). These statistically optimized parameters were then used for the subsequent experimental steps.

**Figure 4:**
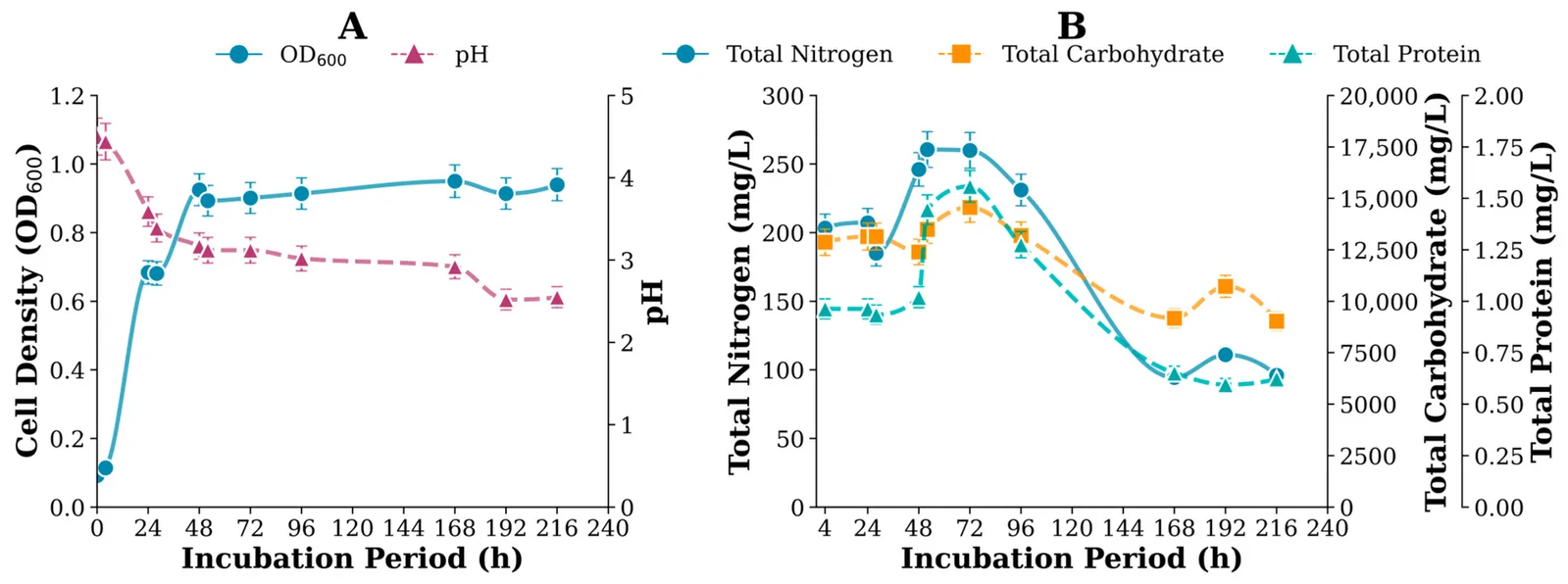
Biochemical composition of f-IA production medium under optimum conditions. (**A**) OD_600_ and pH values during incubation period. (**B**) Extracellular total nitrogen, total carbohydrate and total protein levels during incubation period.

The *S. cerevisiae* cell density and biochemical composition of the production medium under optimized conditions were also investigated (Figure 4). It was observed that the incubation period corresponding to the highest f-IA concentration concurred with the late stationary phase of microbial growth. In addition, the lowest pH value of the medium was detected at this stage, which is thought to result from the accumulation of f-IA and other organic acids produced and secreted into the growth medium by the yeast cells. Throughout the incubation period, based on total carbohydrate and reducing sugar levels approximately 30% of the carbon source was consumed. These findings also suggest that the highest f-IA production was achieved under carbon- and nitrogen-limited conditions after 192 h.

### 2.2. Characterization of the Produced f-IA

**Figure 5:**
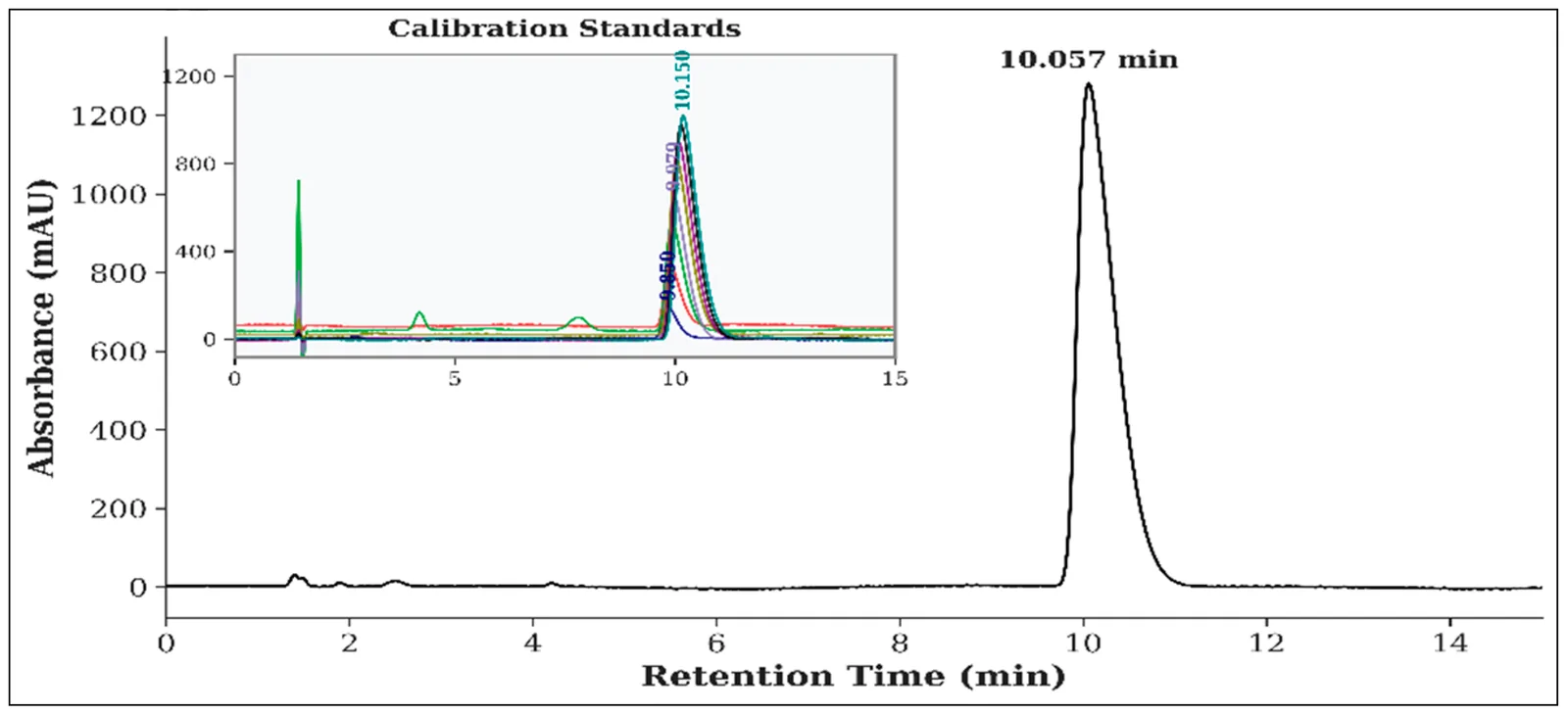
HPLC chromatogram of extracted f-IA and standard c-IA. The inset shows the chromatograms of the calibration standards used for comparison.

**Figure 6:**
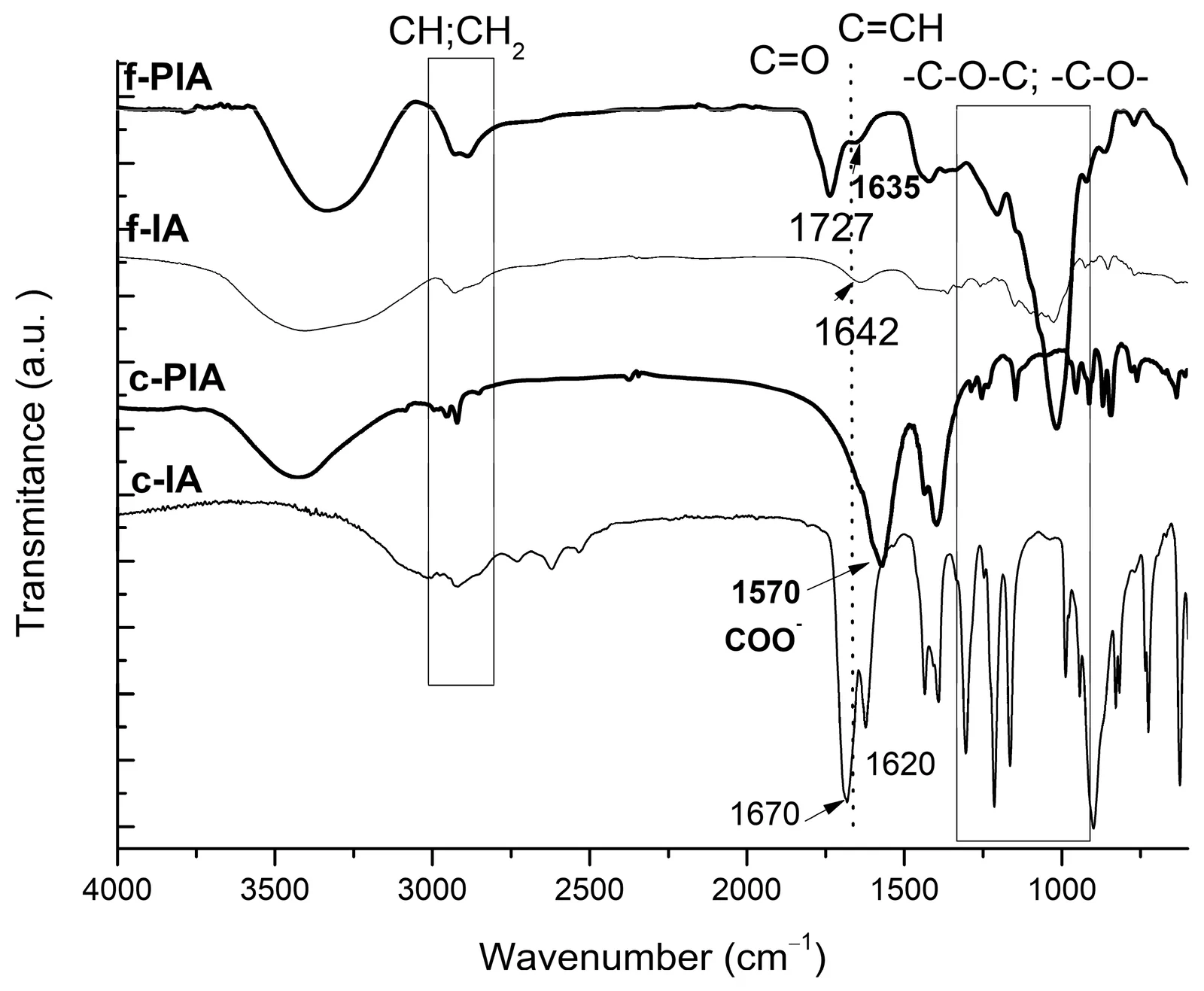
ATR-FT-IR spectra of IA-based samples, including c-IA and f-IA, and their corresponding homopolymers, c-PIA and f-PIA. Prior to polymerization, the c-IA solution was adjusted to pH 6, whereas f-IA was used after purification without further pH adjustment (pH 5).

**Figure 7:**
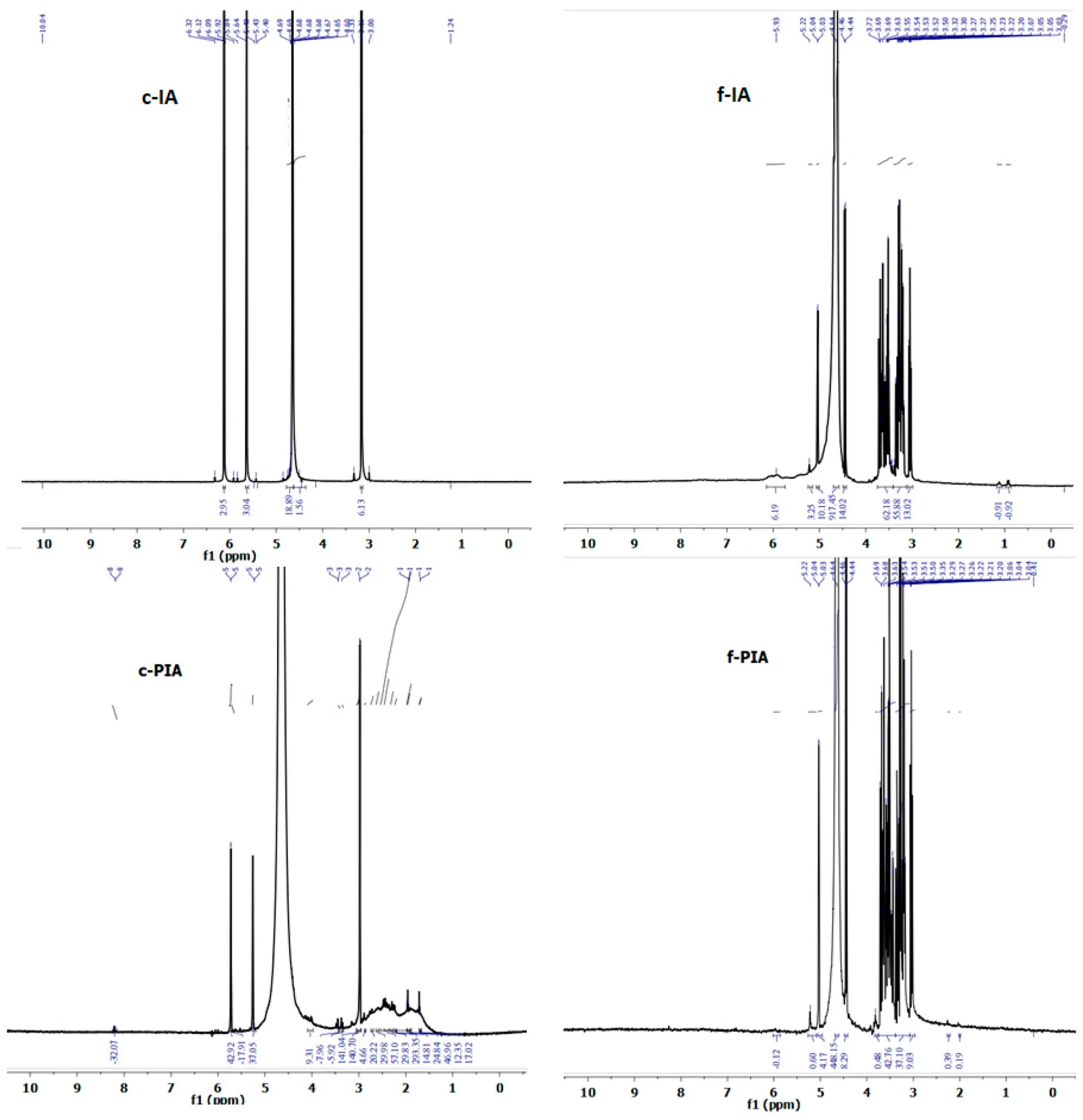
^1^H-NMR spectra of c-IA and f-IA and their obtained homopolymers (c-PIA and f-PIA).

**Figure 8:**
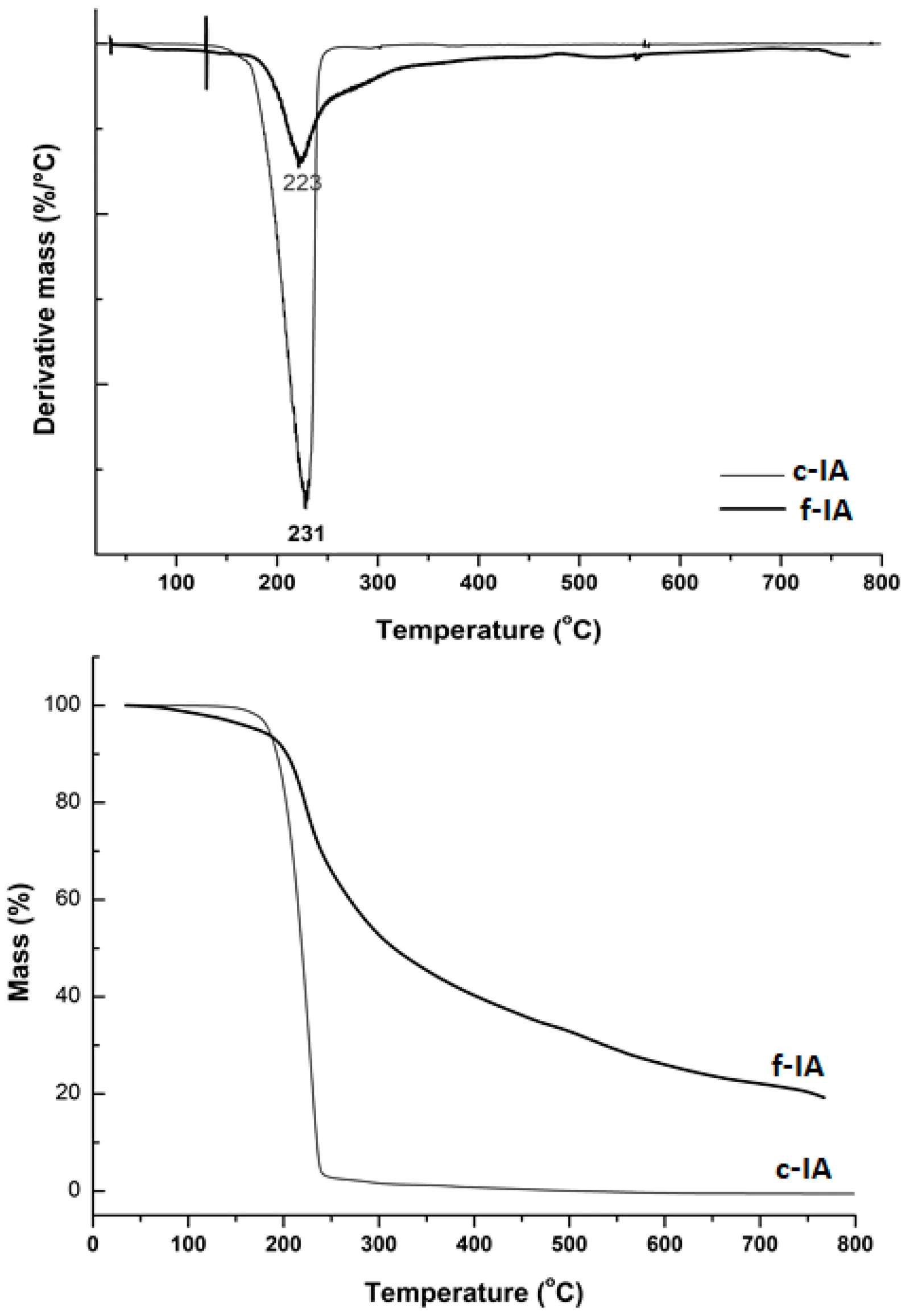
TGA (**bottom**) and corresponding DTG (**top**) curves of c-IA and f-IA. The DTG derivative mass is expressed as % °C^−1^.

After optimization steps, the produced f-IA in optimum conditions was extracted and its chemical characterization was carried out by HPLC, FT-IR, ^1^H-NMR, and TGA/DTG (Figure 5, Figure 6, Figure 7 and Figure 8). Following optimization, the recovered f-IA was quantified using HPLC and yielded a f-IA concentration of 1098 ± 54.12 mg/L. The HPLC chromatogram of the recovered sample exhibited a major peak at a retention time of 10.057 min, closely matching that of the c-IA standard (Figure 5), supporting the identity of the recovered compound.

Following the successful extraction and quantitative analysis of the product, structural characterization was conducted to confirm its molecular identity. FT-IR spectroscopy was employed to verify the functional groups of the f-IA produced under optimum conditions. The spectrum of the extracted sample exhibited a near-perfect overlap with the c-IA standard, strongly validating its structural identity (Figure 6). The broad absorption band observed in the 3300–2500 cm^−1^ region is characteristic of the O–H stretching vibrations of the carboxylic acid groups. The strong peak between 1740 and 1690 cm^−1^ corresponds to the C=O stretching of these carboxylic moieties. Furthermore, the C=C stretching vibration of the alkene group, typically expected in the 1640–1620 cm^−1^ range, was distinctly detected at 1642 cm^−1^. The spectrum also revealed the O–H bending vibrations of the carboxylic acid specifically at 1426.4 cm^−1^ and 1391.9 cm^−1^, which fall perfectly within and adjacent to the expected 1440–1395 cm^−1^ band. Additionally, C–O stretching vibrations were clear in the regions of 1260–1180 cm^−1^ and 1160–1080 cm^−1^. Finally, the =CH_2_ wagging vibration, a definitive signature of the vinyl group in the IA structure, was distinctly observed in the 1000–920 cm^−1^ range. These spectral findings provide complementary evidence supporting the molecular identity of the recovered f-IA and its high chromatographic purity.

^1^H-NMR spectroscopy was used to confirm the molecular structure of the extracted f-IA (Figure 7). The ^1^H-NMR spectrum of f-IA exhibited the characteristic resonances of c-IA, including signals at δ 6.12 and 5.64 ppm corresponding to the two protons of the terminal =CH_2_ group and a resonance at δ 3.16 ppm assigned to the –CH_2_– protons adjacent to the carboxylic acid group and the double bond. The signal at approximately δ 4.69 ppm was attributed to residual HOD/water in the D_2_O solvent. The observed chemical shifts were in close agreement with reported ^1^H-NMR data for f-IA in the HMDB and PubChem databases [20,21], supporting the molecular identity of the f-IA. The spectra of both c-IA and f-IA showed the characteristic vinyl proton resonances of IA.

The thermal behavior of c-IA and f-IA was evaluated by TGA and DTG (Figure 8). The TGA/DTG profile of c-IA showed a sharp and well-defined mass-loss event, with the maximum degradation rate observed at 231 °C. This behavior is consistent with the thermal decomposition reported for neat c-IA, for which decomposition was reported to begin at approximately 136 °C and continue up to 400 °C, with an endothermic decomposition peak at 233.4 °C [22]. The close agreement between the DTG maximum observed for the c-IA sample and the literature value supports the assignment of the major thermal event to the decomposition of c-IA.

In comparison, f-IA showed a broader and less abrupt mass-loss profile, with the principal DTG maximum observed at 223 °C. Although this temperature was approximately 8 °C lower than that of c-IA, both samples exhibited their main degradation event within a similar temperature range. The broader TGA profile of f-IA, together with its considerably higher residual mass at elevated temperatures, clearly distinguishes it from the nearly complete mass loss observed for c-IA. At approximately 800 °C, f-IA retained around 20% of its initial mass, whereas the residual mass of c-IA was negligible. This difference suggests that, in addition to the principal IA-associated degradation, the fermentation-derived sample may contain nonvolatile or thermally persistent constituents that contribute to the gradual mass loss and residual fraction. Such constituents may originate from the fermentation process and/or subsequent purification steps; however, their specific nature cannot be established from TGA/DTG alone.

### 2.3. Characterization of Synthesized PIA (c-PIA and f-PIA)

To investigate the polymerization capability of c-IA and f-IA, both IA samples were subjected to homopolymerization under the polymerization conditions described in Section 4.7. Both obtained polymers (c-PIA and f-PIA) were purified by dialysis for 72 h using dialysis membranes with a molecular weight cut-off (MWCO) of 3.5 kDa and subsequently dried. The isolated gravimetric yield was calculated based on the initial mass of IA used for polymerization according to the following equation: Yield (wt%) = (mass of dried isolated material / initial mass of IA) × 100. The isolated gravimetric yields were approximately 20 wt% for c-PIA and 40 wt% for f-PIA. These values represent isolated mass recovery after purification. The FT-IR and ^1^H-NMR spectra of c-IA, f-IA, c-PIA, and f-PIA are presented in Figure 6 and Figure 7, respectively. FT-IR analysis of c-PIA and f-PIA showed changes in the characteristic absorption bands of c-IA following polymerization. The spectra of c-IA and f-IA exhibited characteristic absorption bands associated with the carboxylic acid groups and unsaturated structure of IA. In the spectrum of c-IA, prominent absorption bands were observed at approximately 1670 and 1570 cm^−1^, while f-IA exhibited a corresponding band at approximately 1642 cm^−1^. Following polymerization, changes in the absorption profiles were observed for both c-PIA and f-PIA, including alterations in the regions associated with the carbonyl and C–O-containing functional groups.

The ^1^H-NMR spectra of both c-IA and f-IA exhibited the characteristic vinyl proton resonances of IA, including signals at δ 6.12 and 5.64 ppm corresponding to the two protons of the terminal =CH_2_ group. Following polymerization, these characteristic vinyl proton signals were substantially reduced or no longer clearly resolved in both c-IA and f-IA, while broader and more complex resonances were observed predominantly in the aliphatic region.

## 3. Discussion

Among bio-based platform chemicals, IA has attracted considerable attention due to its unique chemical structure and broad industrial applicability [1]. Its unsaturated dicarboxylic structure, containing both a vinyl group and two carboxyl groups, makes it a versatile building block for the production of bio-based polymers, coatings, adhesives, plasticizers, and other value-added materials. In recent years, various fungi, yeast and bacteria types have been explored to overcome high and safe IA production drawbacks. Overall, strain selection focuses on three parameters: (1) high yield and productivity, (2) tolerance to acidity, and (3) the ability to grow on cheap, renewable feedstocks [23,24]. Beyond production performance, the safety status of the production organism is another important consideration, as the use of organisms classified as non-pathogenic can simplify the production process and broaden the potential applications of the final product. Therefore, the advantages of non-pathogenic microorganisms have gained increasing attention, and *S. cerevisiae* was evaluated in this study as a potential producer for IA. Previous studies have also demonstrated the potential of yeast species for IA production. In a study using *S. cerevisiae*, an IA titer of 0.851 g/L was reported [25]. Similarly, *Pichia kudriavzeii* was metabolically engineered for IA production, and when 20 g/L glucose was used as the carbon source, a maximum IA titer of 1.232 g/L was obtained [26]. These findings further support the potential of yeast-based systems for IA production and provide a useful reference for evaluating the production performance obtained in the present study.

Efficient IA production relies on the integrated optimization of some fermentation parameters such as carbon concentration, pH and metal ion concentration. In the present study D-glucose was evaluated as a carbon source in different concentrations. While glucose is widely recognized as a highly effective and standard carbon source, lignocellulosic biomass has also gained attention due to its low cost and availability [14]. However, these substrates require pretreatment and hydrolysis, which may introduce inhibitors, reduce overall productivity and increase production cost. Therefore, glucose was selected as the carbon source for this study due to its consistently higher performance in f-IA production. This finding is consistent with previous studies in which different pure sugars, including glucose, fructose, and sucrose, were evaluated as carbon sources, with the highest IA production being obtained when 50 g/L glucose was used [27]. In addition, IA production by *A. terreus*, one of the well-established IA-producing organisms, has been investigated using an initial glucose concentration of 120 g/L, further highlighting the importance of carbon availability for IA production [28].

IA biosynthesis is tightly linked to central carbon metabolism and proceeds via the tricarboxylic acid cycle. Glucose is initially catabolized through glycolysis to pyruvate in the cytosol. Pyruvate is subsequently transported into the mitochondria and converted to acetyl-CoA, which condenses with oxaloacetate via citrate synthase to form citrate. Citrate is then isomerized to cis-aconitate by mitochondrial aconitase. Following its transport to the cytosol, cis-aconitate is decarboxylated by cis-aconitate decarboxylase to yield itaconate. The final product is exported across the plasma membrane via specific transport systems [29]. Therefore, increasing the glucose concentration may increase the availability of carbon for flux through the citrate cycle, potentially promoting greater IA production. The findings of the present study are consistent with this possibility, as the highest f-IA yield was obtained at the highest glucose concentration evaluated (Figure 1A). Because increasing the initial carbon source concentration while maintaining the nitrogen concentration results in an increased C/N ratio, the higher f-IA production observed under these conditions may also be associated with the effect of carbon-to-nitrogen balance on metabolic flux. Niehoff et al. (2023) identified the C/N ratio as a key factor for IA production, supporting the importance of this parameter in determining the balance between biomass formation and organic acid accumulation [27].

Additionally, nutrient limitation—particularly nitrogen restriction—is commonly applied to redirect carbon flux from biomass formation toward product synthesis, with high C/N ratios promoting IA accumulation [30]. Previous studies in different IA-producing microorganisms have also associated nitrogen limitation with the onset of IA production. For example, in Ustilaginaceae, the onset of nitrogen limitation coincided with the transition from exponential growth to a nitrogen-limited production phase, during which IA production was induced [27]. Similarly, nitrogen-limited conditions increased IA production in engineered *Corynebacterium glutamicum*, suggesting that nitrogen availability can affect IA production [31]. However, these studies were performed in different microbial hosts, and therefore the observed relationship between nitrogen limitation and IA production should not be considered as direct evidence of a causal effect in *S. cerevisiae*. In the present study, higher f-IA production was observed after 168 h of cultivation, when the nitrogen level in the growth medium became limiting and the C/N ratio increased (Figure 4B). This period also coincided with the stationary phase of *S. cerevisiae* growth (Figure 4A), which may suggest a relationship between nitrogen limitation, the stationary phase, and increased f-IA production. Nitrogen limitation has been proposed to reduce further biomass formation and favor product synthesis; however, other factors, such as oxygen availability and precursor supply, may also affect IA production [32]. Therefore, the results obtained in the present study suggest that nitrogen limitation may contribute to the increased f-IA production observed during the stationary phase. However, the present results demonstrate an association between nitrogen availability and IA accumulation rather than a direct causal relationship, and they do not demonstrate that nitrogen limitation alone is responsible for f-IA production. Further experiments would therefore be required to confirm the specific role of nitrogen limitation in IA production.

In the present study, the highest f-IA concentration was likewise reached after the onset of nitrogen limitation at 192 h (Figure 4B). The conversion of sugars into IA leads to a net accumulation of energy equivalents, including NADH and ATP; therefore, nitrogen limitation may contribute to maximum IA production [27,33]. When sufficient carbon and nitrogen levels are available, cellular metabolism is generally considered to be preferentially directed toward biomass formation. Consistent with this metabolic principle, increasing the initial carbon source concentration in the present study altered the C/N ratio, and the highest f-IA production was observed under conditions associated with a higher C/N ratio (Figure 1A).

Among operational parameters, pH control is one of the most critical factors [28]. The pH of the fermentation medium governs the distribution of IA species and can influence both fermentation performance and downstream processing. While lower pH values favor the protonated form of IA, reducing acid consumption and simplifying product recovery, excessive acidification can impair microbial metabolism by increasing cellular energy demand and product inhibition. Therefore, careful optimization of fermentation pH is essential to balance production efficiency with process economics. When the pH becomes too low, the undissociated acid may enter the cells and increases energy loss, which can reduce productivity. Based on the dissociation constants of IA (pKa = 3.85–5.55), a simple calculation suggests that less than 1% of IA remains in the desired undissociated form at pH 5.6. To ensure that most of the produced IA remains undissociated, the pH would have to be lowered to below approximately 2.3. However, such highly acidic conditions are generally unsuitable for the growth of most microbial hosts [26,27]. Among the initial pH values evaluated in this study, pH 5.6 provided moderately favorable conditions for the growth of the microorganism. Although pH 3.0 promoted f-IA production, it may have imposed considerable stress on microbial growth. In contrast, an initial pH of 4.5 provided the most suitable ionic environment, which was associated with efficient microbial growth and enhanced f-IA production, thereby representing suggesting a suitable balance between biomass formation and metabolite synthesis (Figure 1B). In the present study, the optimum initial pH was determined to be 4.5 (Figure 1B), while the pH decreased to approximately 2.5 during the incubation period in which the highest f-IA production was observed (Figure 4A). This observation suggests that an excessively low initial pH may not be necessary for IA production and that maintaining a moderately acidic initial condition may be important to allow sufficient adaptation and growth of the microorganism. In addition, very low initial pH values may impose weak-acid stress on microbial cells [27].

A similar spontaneous acidification pattern has been reported in other yeast-based IA production systems. Sun et al. (2020) reported an initial pH of 6.1 in the production medium, which decreased to 4.7 during the first 6 h without external pH adjustment and subsequently remained relatively stable at approximately 3.9 throughout the remainder of the process [26]. In the present study, the pH similarly decreased to 3.59 within the first 24 h and subsequently remained within the range of 3.15–2.50. The maintenance of the pH within the range of approximately 2–3 during IA production may have contributed positively to IA accumulation, consistent with previous findings indicating that acidic conditions can favor IA production [28].

Furthermore, the effect of metal ion concentration, specifically calcium, on f-IA production was also investigated as a critical fermentation parameter. While metal ions like calcium are generally essential for yeast cell wall integrity and various enzymatic activities, our findings indicated that the addition of CaCI_2_ did not enhance f-IA accumulation. In fact, both the kinetic evaluations (Figure 1C) and the statistical analysis revealed that CaCl_2_ concentration was the least influential parameter among the evaluated factors. The maximum f-IA titer was achieved in the medium without any CaCl_2_ supplementation (0 g/L). This phenomenon may be attributed to the possibility that stress-inducing conditions—such as the limitation of specific metal ions—can restrict excessive biomass proliferation, thereby potentially favoring the accumulation and secretion of organic acids like IA as a physiological response. In yeast cells, the absence of calcium may affect biochemical and signaling mechanisms, potentially leading to changes in cell cycle progression, metabolic stress responses, and cell wall integrity. In the absence of calcium, the ability of cells to efficiently utilize carbon sources may also be reduced [34,35]. However, the physiological mechanism underlying the observed effect of calcium limitation on IA production was not directly investigated in this study and therefore cannot be confirmed based on the present experimental data. From potential industrial and large-scale perspective, achieving optimal production without the need for additional CaCl_2_ supplementation may offer an economic advantage by simplifying the medium composition and reducing overall process costs.

Trace metal ions significantly affect IA production, primarily through their roles in enzyme activity and metabolic flux distribution [18]. While calcium compounds typically contribute to fermentation stability and pH regulation during microbial organic acid production [36], deviations from optimal metal concentrations often may induce metabolic shifts. Previous studies have well-documented that nutrient limitation or heavy ionic stress limits cellular biomass increase, thereby potentially favoring the accumulation of organic acids as a physiological overflow response [37]. In the present study, CaCl_2_ supplementation did not enhance f-IA production, and the highest production was obtained in the absence of additional CaCl_2_ This finding suggests that additional CaCl_2_ was not required to improve IA accumulation under the investigated fermentation conditions. The absence of additional CaCl_2_ may have contributed to the enhanced f-IA accumulation observed under these conditions; however, the underlying physiological mechanism was not directly investigated in the present study. Under the conditions investigated in this study, CaCl_2_ supplementation did not enhance f-IA production, and the highest f-IA concentration was obtained without additional CaCl_2_ supplementation.

Following optimization, the recovered f-IA, corresponding to a yield of approximately 0.3795 g/g consumed glucose, was further characterized using complementary analytical techniques to confirm its molecular identity and purity, with the results compared against those of the corresponding commercial standard and literature data. HPLC analysis showed a single major peak at 10.057 min, which closely matched the retention time of the c-IA standard, supporting the identity of f-IA and indicating high chromatographic purity based on HPLC analysis. It should also be noted that the spectrophotometric assay based on bromination used for monitoring f-IA production may be less selective than HPLC analysis. Since fermentation broths contain amino acid compounds, such as the produced protein, this contributes to the measured absorbance and could lead to an overestimation of the apparent IA concentration. In contrast, the isolated f-IA was further verified by HPLC based on chromatographic separation and its characteristic retention time.

FT-IR analysis further confirmed the characteristic functional groups of IA, including the C=C stretching vibration at 1647.7 cm^−1^ and the characteristic =CH_2_ vibrations in the 1000–920 cm^−1^ region, in agreement with the expected absorption bands of IA. The ^1^H-NMR profile of the recovered f-IA was consistent with the expected molecular structure of IA, supporting its molecular identity. The overall resonance pattern showed good agreement with that of the c-IA standard and with previously reported literature data, further confirming the successful recovery of f-IA. Minor differences in chemical shifts may arise from variations in sample concentration, solvent environment, and pH. Taken together, the HPLC, FT-IR, and ^1^H-NMR results provide complementary evidence confirming the successful recovery of f-IA with the expected chemical structure and high chromatographic purity.

The thermal behavior of the recovered f-IA was subsequently evaluated by TGA/DTG. Allan et al. (1987) reported that neat IA was thermally stable up to approximately 136 °C, followed by pyrolytic decomposition extending to approximately 400 °C [38]. In their DSC measurements, the melting/fusion of IA occurred at 171.3 °C, followed by an endothermic decomposition event at 233.4 °C. Thus, the DTG maximum observed for c-IA in the present study (231 °C) is highly consistent with the decomposition-related thermal event reported by Allan et al. (1987) (233.4 °C), supporting the assignment of the major mass-loss event to IA decomposition [38]. They also reported the TG/DTG measurements at a heating rate of 6 °C min^−1^ in static air over 20–500 °C, whereas the present measurements were conducted under the conditions described in the Materials and Methods section; therefore, differences in experimental conditions should be considered when making direct comparisons [38].

The f-IA sample exhibited a principal DTG maximum at 223 °C, approximately 8 °C below that of c-IA and 10 °C below the decomposition-related peak reported by Allan et al. (1987) [38]. Despite this modest shift, the proximity of the principal degradation temperatures indicates that the dominant thermal degradation event of f-IA occurs within the same temperature region as that of c-IA. However, f-IA displayed a considerably broader TGA profile and a substantially higher residual mass at high temperature than c-IA, with approximately 20% residual mass remaining at 800 °C. This difference is consistent with the more complex composition that can be associated with f-IA. In this regard, the recent review by Jašek et al. (2026) emphasizes the biotechnological origin of IA and notes that biotechnologically produced IA may retain residual glucose from the fermentation process if purification is incomplete [39]. Therefore, the broader degradation profile and higher residual mass observed for f-IA may reflect the contribution of residual fermentation-derived or other thermally persistent nonvolatile constituents. Accordingly, the TGA results do not support a conclusion regarding the overall chemical purity of f-IA; rather, they complement the HPLC results by demonstrating differences in the thermal behavior and composition of the recovered f-IA.

Importantly, the recovered f-IA was not only characterized as a fermentation product but was also evaluated for its ability to undergo polymerization. Comparison of the ^1^H-NMR spectra of c-IA and f-IA with those of their corresponding polymers, c-PIA and f-PIA, revealed substantial changes in the proton resonance profiles following polymerization. In particular, the characteristic vinyl proton signals of IA were markedly reduced or no longer clearly resolved in both c-PIA and f-PIA, while broader and more complex signals were observed in the aliphatic region. These changes are consistent with the consumption of the C=C double bond during polymerization and the formation of the polymer backbone. However, the observed changes in the ^1^H-NMR spectra provide evidence for consumption of the IA C=C double bond but do not establish the molecular weight or molecular-weight distribution of the resulting polymers. Molecular-weight characterization by techniques such as gel permeation chromatography or size-exclusion chromatography was not performed in the present study. The 3.5 kDa MWCO dialysis membrane was used for purification of the polymer products and could not be used to interpret the molecular weight, as the dialysis only separated the species based on their ability to pass through the membrane. Even though the starting pH of the c-IA solution (pH 6) differed by approximately one pH unit from that of the recovered f-IA (pH 5), and this difference may influence the ionization state of IA and potentially affect radical polymerization behavior, both samples were subjected to the same polymerization procedure. Therefore, this comparison should be interpreted as a proof-of-concept assessment of the polymerization capability of f-IA rather than as a strictly controlled comparison at identical pH. The comparable overall spectral changes observed for c-PIA and f-PIA indicate that the f-IA retained its ability to undergo polymerization under the applied conditions. Thus, the polymerization experiment provides an initial proof of concept for the use of the newly obtained f-IA as a renewable monomer for further polymer synthesis.

Overall, the results demonstrate the feasibility of producing f-IA using *S. cerevisiae* BY4742 under the investigated conditions. Furthermore, the demonstrated polymerization ability of the recovered f-IA provides a proof of concept for its potential use as a renewable monomer for further polymer synthesis. However, the relatively long fermentation period required to reach the maximum IA concentration (192 h) and the moderate IA concentration obtained (~1.1 g/L) indicate that the present process should be considered a proof of concept rather than an optimized production process. Further studies addressing strain improvement, fermentation kinetics, substrate utilization, productivity, and process intensification are required before the industrial applicability of this production platform can be evaluated.

## 4. Materials and Methods

### 4.1. Materials

Analytically grade D-glucose, CaCI_2_, yeast extract, peptone, agar, bromine, *n*-butanol, ortho-phosphoric acid and IA (99%), and LiChrosolv methanol were obtained from Sigma-Aldrich, Schnell Dorf, Germany. The initiator system (ammonium persulfate—APS, H_2_O_2_—35% solution) was purchased from Sigma-Aldrich, Hamburg, Germany. NaOH was purchased from Sigma-Aldrich (Darmstadt, Germany). A dialysis membrane with a molecular weight cut-off of 3.5 kDa, obtained from Scienova GmbH (Jena, Germany), was used for purification.

### 4.2. Microorganism Growth Conditions

To maintain culture viability, *S. cerevisiae* BY4742 was grown on yeast extract-peptone-D-glucose (YPD) agar medium (pH 5.6) [40]. Stock cultures were incubated at 30 °C for 5 days and subsequently stored at 4 °C for further use.

### 4.3. IA Production Under SmF Conditions

IA production was implemented under SmF conditions using 100 mL Cooper and Paddock’s liquid medium with different D-glucose concentrations (1, 3 and 10%, *w*/*v*) in 250 mL Erlenmeyer flasks [40]. A 5-day-old *S. cerevisiae* culture grown on YPD agar was aseptically suspended in water until an optical density (OD_600_) of 0.8–1.0 was reached, and 5% (*v*/*v*) of the suspension was inoculated into each flask. The cultures were incubated at 30 °C, 100 rpm for 9 days. After determination of optimum carbon source concentration, optimum pH value (at 3, 4.5 or 5.6) was investigated. Lastly, effects of different CaCI_2_ concentrations (0, 0.01, 0.05 g/L) on f-IA production were analyzed.

### 4.4. Experimental Design

Carbon concentration, pH, and CaCl_2_ concentration were selected as important process factors based on previous studies on microbial IA and organic acid production. Therefore, these three independent variables were optimized using BBD. These independent variables were tested at three levels: high (+1), middle (0), and low (−1). The variable levels and the full experimental design are shown in Table 2 and Table 3. A second-order quadratic model was then used to predict the optimum production conditions. The model is shown in Equation (1):

Y = β_0_ + ∑β_i_X_i_ + ∑β_ii_X_i_^2^ + ∑β_ij_X_i_X_j_ (1)

In this equation, Y represents the predicted response, which is f-IA concentration in mg/L. X_i_ and X_j_ represent the coded independent variables. β_0_ is the intercept of the model. β_i_ shows the linear effect, β_ii_ shows the quadratic effect, and β_ij_ shows the interaction effect between two variables. ANOVA was used to evaluate the statistical significance and adequacy of the model.

### 4.5. Analysis of Production Medium Components

Microbial growth was determined based on OD_600_ values measured throughout the incubation period. The pH level of the liquid phase in the production medium was monitored. The production medium was centrifuged (5000 rpm, 10 min, 4 °C) and the supernatants were used for subsequent tests. The levels of total carbohydrates, total nitrogen, total protein, and reducing sugars in the production medium were determined using the phenol–sulfuric acid [41], phenol–hypochlorite [42], Bradford (1976) [43], and dinitrosalicylic acid [44] methods, respectively.

The produced f-IA concentration was measured spectrophotometrically [45]. The reported IA concentrations refer to the volume of the fermentation broth, specifically the cell-free supernatant, and therefore represent the IA concentration in the fermentation broth (mg/L). Production medium supernatant (1 mL) was mixed with 0.3 mL bromine reagent and the pH was adjusted to 1.2 with 1 M HCl. The final mixture was incubated at room temperature for 15 min and absorbance was measured at 385 nm. c-IA was used as standard.

### 4.6. Isolation and Characterization of the Produced f-IA

The pH of the fermentation medium was adjusted to 1.00 to facilitate the protonation of f-IA and its transfer into the solvent phase for isolation. Subsequently, equal volume of *n*-butanol was added, and the mixture was incubated at room temperature under agitation at 200 rpm for 4 h. Following incubation, phase separation was achieved by centrifugation at 5000 rpm for 15 min. Since n-butanol forms the upper organic phase, the lower aqueous phase was separated [46]. Thereafter, the pH of the aqueous phase was increased by titration using 0.1 M NaOH, enabling the re-extraction of f-IA into the aqueous phase. Finally, f-IA was isolated from the aqueous phase by crystallization.

#### 4.6.1. HPLC Analysis

The produced f-IA was analyzed by using an Agilent 1260 HPLC system. The system was equipped with a Variable Wavelength Detector and a Chrompack C18 column with a length of 150 × 4.6 mm, Santa Clara, CA, USA. Mobile phase was 98% acidified ultrapure water with orthophosphoric acid at a pH 2.3 and 2% methanol (*v*/*v*). The column’s temperature was set at 30 °C, the flow rate was 1 mL/min, the UV detection was set at 210 nm and the injected volume was 20 µL. For the calibration curve, stock solutions of c-IA were used. The peak area correlated linearly within the concentration range (R^2^ = 0.9869; y = 16.47867x + 32.213712).

#### 4.6.2. FT-IR Analysis

The FT-IR spectra of the produced and isolated f-IA, c-IA, f-PIA and c-PIA were recorded using an ATR-FT-IR instrument (Perkin Elmer Spectrum Two, Headquarters 710 Bridgeport Ave, Shelton, CT 06484, USA). Spectra were recorded at room temperature in the range of 4000–500 cm^−1^.

#### 4.6.3. ^1^H-NMR Analysis

For NMR spectroscopy, the extracted f-IA, c-IA, f-PIA and c-PIA were dissolved in D_2_O. ^1^H-NMR spectrum was recorded on a MERCURY Plus-AS 400 spectrometer (Varian, Palo Alto, CA, USA) at 400 MHz and 30 °C. The chemical shifts were expressed in ppm.

#### 4.6.4. TGA

The thermal decomposition behavior of the extracted f-IA and c-IA, were analyzed by TGA (Perkin Elmer, Headquarters 710 Bridgeport Ave, Shelton, CT 06484, USA). Approximately 5 mg of the dried samples were analyzed under a nitrogen atmosphere over a temperature range of 35–800 °C at a heating rate of 15 °C/min. The TGA and DTG curves were recorded to evaluate the mass-loss behavior and the corresponding thermal decomposition temperature of the recovered compounds.

### 4.7. Synthesis Procedure of PIA (c-PIA and f-PIA)

To evaluate the polymerization ability of both c-IA and f-IA were subjected to polymerization using the same polymerization procedure. For c-IA, 2 g of IA was dissolved in 20 mL of hot distilled water, and the solution was adjusted to pH 6 using a 20 wt% NaOH solution. The f-IA, which had a pH of approximately 5 following recovery was used without further pH adjustment. The two IA solutions were separately transferred into three-neck flasks and preheated to 72 °C to facilitate activation of the initiator system. APS (9 mg) and approximately 0.03 g of 35% H_2_O_2_ were then added to each reaction mixture. Based on the amount of APS used, the molar ratio of IA to APS was approximately 390:1. Subsequently, the reaction temperature was increased to 85 °C, and polymerization was carried out under continuous magnetic stirring at 85 °C for 72 h.

Following completion of the polymerization reactions, the resulting c-PIA and f-PIA products obtained from c-IA and f-IA, respectively, were separately purified by dialysis for 72 h using dialysis membranes with a MWCO of 3.5 kDa.

## 5. Conclusions

This study demonstrates that *S. cerevisiae* BY4742 has potential as a microbial platform for the production of bio-based IA under SmF conditions. The optimized fermentation process enabled the recovery of f-IA with confirmed molecular identity and high chromatographic purity, as supported by complementary spectroscopic and chromatographic analyses. Importantly, the f-IA retained its polymerization ability, as demonstrated by the changes in the characteristic vinyl proton signals following polymerization. These findings provide a basis for further optimization of *S. cerevisiae*-based IA production and its application in the development of bio-based polymeric materials. Nevertheless, the relatively long fermentation period (192 h) and the f-IA concentration obtained (approximately 1.1 g/L) indicate that the present findings should be regarded as a proof of concept rather than evidence of industrial competitiveness. Further improvements in fermentation performance and process productivity are required before the industrial applicability of this approach can be evaluated.

## Figures and Tables

**Table 1:** Statistical analysis results of the BBD for f-IA production.

Model Term	Coefficient Estimate	Standard Error	t-Value	*p*-Value
Intercept	313.190	185.231	1.69	0.1517
X_1_ (Carbon Concentration)	50.883	4.351	11.69	<0.0001 *
X_2_ (pH)	−42.352	24.435	−1.73	0.1436
X_3_ (Ca^2+^ Concentration)	−2553.823	1364.079	−1.87	0.1201
X_1_^2^	17.984	3.831	4.69	0.0054 *
X_2_^2^	−105.727	27.755	−3.81	0.0125 *
X_3_^2^	267,150.590	128,005.600	2.09	0.0912
X_1_X_2_	4.403	4.359	1.01	0.3589
X_1_X_3_	162.432	198.695	0.82	0.4508
X_2_X_3_	794.568	870.961	0.91	0.4035

* Statistically significant (*p* < 0.05).

**Table 2:** Levels of independent variables used in BBD.

Factor Code	Independent Variable	Unit	Low Level (−1)	Middle Level (0)	High Level (+1)
X1	Carbon concentration	%	1	3	10
X2	pH	pH	3	4.5	5.6
X3	CaCl_2_ concentration	g/L	0	0.01	0.05

**Table 3:** Experimental design and corresponding results of BBD.

Treatment	X1 (%)	X2	X3 (g/L)	IA (mg/L)
1	1	5.6	0.01	443.5
2	3	5.6	0.01	223.5
3	10	5.6	0.01	858.5
4	10	3	0.01	773.5
5	10	4.5	0.01	968.5
6	10	4.5	0	1133.5
7	10	4.5	0.05	1128.5
8	1	3	0.05	390.5
9	10	3	0.05	785
10	1	5.6	0.05	450
11	10	5.6	0.05	880.5
12	1	4.5	0	660.5
13	1	4.5	0.01	590
14	3	3	0	470
15	3	5.6	0.05	250.5

## Data Availability

The data presented in this study are available on request from the corresponding author.

## Abbreviations

The following abbreviations are used in this manuscript:

IA	Itaconic acid
c-IA	Commercial itaconic acid
f-IA	Fermentation-derived IA
PIA	Poly(IA)
c-PIA	Poly(commercial IA)
f-PIA	Poly(fermentation-derived IA)
SmF	Submerged fermentation
BBD	Box–Behnken Design
RSM	Response Surface Methodology
YPD	Yeast extract-peptone-D-glucose
